# Implementation of Endogenous and Exogenous Mesenchymal Progenitor Cells for Skeletal Tissue Regeneration and Repair

**DOI:** 10.3390/bioengineering7030086

**Published:** 2020-08-04

**Authors:** Salomi Desai, Chathuraka T. Jayasuriya

**Affiliations:** Department of Orthopaedics, Warren Alpert Medical School of Brown University and the Rhode Island Hospital, Providence, RI 02903, USA; salomi_desai@brown.edu

**Keywords:** skeletal tissue repair, regeneration, stem cell, progenitor cell, bone, cartilage, fibrocartilage, tendon, ligament

## Abstract

Harnessing adult mesenchymal stem/progenitor cells to stimulate skeletal tissue repair is a strategy that is being actively investigated. While scientists continue to develop creative and thoughtful ways to utilize these cells for tissue repair, the vast majority of these methodologies can ultimately be categorized into two main approaches: (1) Facilitating the recruitment of endogenous host cells to the injury site; and (2) physically administering into the injury site cells themselves, exogenously, either by autologous or allogeneic implantation. The aim of this paper is to comprehensively review recent key literature on the use of these two approaches in stimulating healing and repair of different skeletal tissues. As expected, each of the two strategies have their own advantages and limitations (which we describe), especially when considering the diverse microenvironments of different skeletal tissues like bone, tendon/ligament, and cartilage/fibrocartilage. This paper also discusses stem/progenitor cells commonly used for repairing different skeletal tissues, and it lists ongoing clinical trials that have risen from the implementation of these cells and strategies. Lastly, we discuss our own thoughts on where the field is headed in the near future.

## 1. Introduction

Repair/regeneration of damaged tissue is fundamental in the maintenance of normal homeostasis and it is fine-tuned at the cellular and molecular levels. The process of wound healing is highly conserved across the animal kingdom from some of the most primitive organisms, such as hydra [1] and amoeba [2], to advanced organisms like mammals [3,4]. However, the regenerative capabilities of advanced vertebrates are comparatively limited. In humans, certain tissue types, such as blood vessels and epidermis or the gastrointestinal track, can repair within hours, whereas in parts of the nervous system and skeletal system, repair and remodeling may take several weeks to months [5]. 

Musculoskeletal tissue is on the front line of exposure to injury in any active individual. It comprises nearly 40% of the total body mass and it is vital for body movements, which are tightly regulated by the coordinative efforts of its different components, such as bone, cartilage, muscle, ligament, and tendon. Skeletal tissue injuries occur by various means, which include, but are not limited to: overuse (i.e., wear and tear), trauma (i.e., accidental/sport injuries), and degenerative diseases (i.e., muscular dystrophy, osteoarthritis, etc. [6,7]). In most tissues, wound healing begins with the formation of a blood clot [8,9], followed by the recruitment of mesenchymal stem cells (MSCs). Studies have shown that MSCs are integral to the repair process as they help replace damaged tissue by differentiating and directly replacing damaged tissue [10], as well as by mediating indirect paracrine actions that regulate the migration and behavior of mature cells to promote healing [11,12]. These paracrine effects are mediated by factors secreted into the extracellular space by MSCs that are collectively referred to as the secretome, which regulates immunomodulation, angiogenesis, migration, cell proliferation/survival features. For further reading on the MSC secretome and its influences on tissue healing, please refer to Daneshmandi et al., 2020 [13]. 

**Figure 1:**
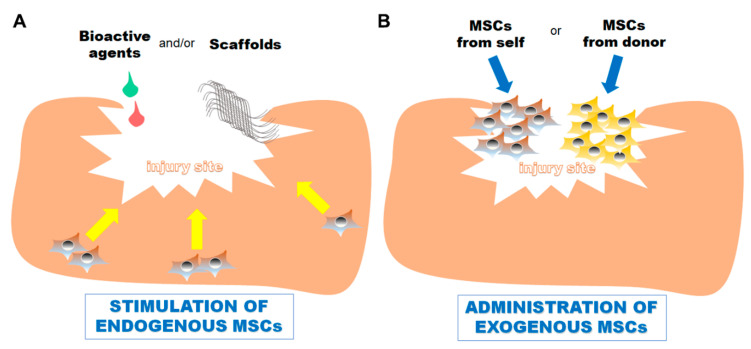
Conceptual diagrams of the two main approaches used to stimulate skeletal tissue healing and repair by sequestering the help of stem/progenitor cells. (**A**) Biomaterials/scaffolds and/or bioactive agents, such as growth factors, chemokines, and small molecules are placed at the site of injury to stimulate the migration and differentiation of endogenous native mesenchymal stem cells for aiding in the repair process. (**B**) Cells are physically administered exogenously, from one anatomical location of the patient to the site of injury, or from a donor, to aid in the repair process. This can be done with or without a scaffold or biomaterial to hold the newly administered cells in place.

The skeletal system consists of different tissues which exist in varying micro-environments that inherently have, or do not have, access to specific resources [14]. Due to these complexities, not all tissues have the same level of access to MSCs, hence factoring into why repair efficacy can vary dramatically between different skeletal tissues. In efforts to account for this disparity, different cell-based approaches have been implemented in order to jumpstart the repair process, including administering (or physically relocating) exogenous MSCs as biologic therapies to injury sites, or administering chemokines, growth factors, scaffolds (and combinations) to stimulate activation and migration of native endogenous MSCs to these locations (Figure 1). Surgical procedures (such as tibial microfracture surgery for knee cartilage focal defect repair) have also been developed to facilitate the migration of native MSCs from one tissue to another (i.e., bone to cartilage), without having to physically relocate the cells, in order to facilitate repair. Here, we will review the repair efficacies of both “endogenous” and “exogenous” cell-based repair approaches as they have been reported in different pre-clinical models. We will also touch on the limitations of these strategies.

## 2. Common Progenitor/Stem Cells Utilized for Skeletal Tissue Repair

Stem cell-based therapies have gained great interest from scientific communities in the last two decades, not only for their dynamic potential to treat infectious diseases and cancers, but also for their ability to stimulate tissue healing. Strategies to enhance healing by mimicking the natural cellular processes that occur during skeletal development are actively being explored. Most notably, transplantation of tissue specific progenitor cells or stimulating the recruitment of endogenous/native progenitors are approaches that are utilized to enhance healing of skeletal tissues in preclinical models [15,16,17,18,19]. The self-renewal capacities of skeletal tissues diminish with aging. This is partly attributed to the reduced abundance and efficiency (i.e., loss of proliferative capacity and/or differentiation potential) of native adult stem cells that are normally involved in repairing these tissues. The importance of recruiting and/or administrating cells with the greatest potential to rebuild the specific skeletal tissue in question have been highlighted in previous reviews [20,21,22,23,24].

**Figure 2:**
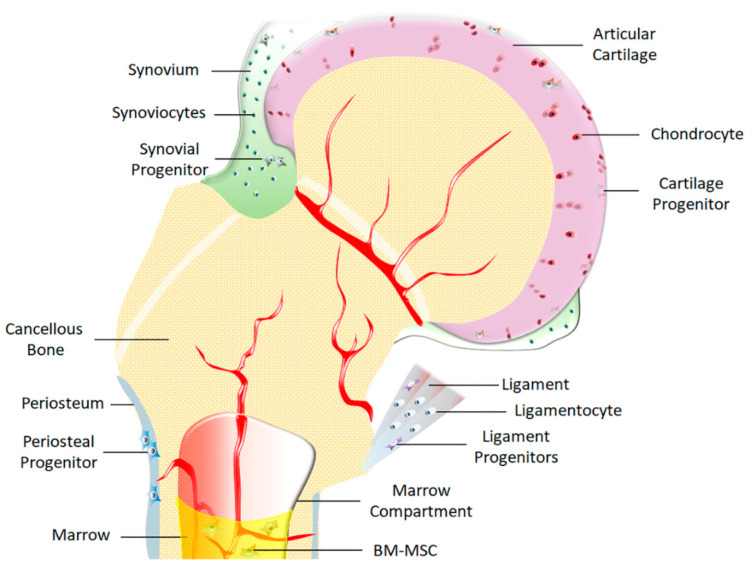
Diagram depicting various sources of Mesenchymal Stem Cells (MSCs) and their cellular microenvironments of adjacent skeletal tissues that make up a joint. These include MSCs from synovium, cartilage, bone marrow, periosteum, and ligament/tendon. Additionally, MSCs from adipose tissue such as fat pad (not depicted here) are also present. This represents how each tissue is not a closed system but rather that they are constantly in the direct proximity of MSCs from adjacent tissues.

Human clinical trials involving stem/progenitor cells for the treatment of musculoskeletal injuries are increasing [25,26,27,28,29]. Many of these trials involve somatic (adult) stem cells, with mesenchymal stem/progenitor cells being the most commonly used variety. MSCs are essential for the development and repair of the skeletal system—from embryonic bone formation to adult fracture healing and remodeling [30]. MSCs are heterogeneous populations of multipotent cells residing in adult tissues [30,31]. These cells are scattered throughout the skeletal system and they aid in the restoration of damaged tissue (Figure 2).

MSCs can be isolated from marrow [32], periosteal bone [33,34], cartilage [35,36,37], fibrocartilage [38,39], adipose tissue [40], tendon [41], ligament [42,43,44], and synovium [45]. MSCs isolated from these different tissues may vary in phenotype, morphology, differentiation, and proliferation capacity, but the consensus is that they all possess similar characteristics to bone marrow derived stromal cells (BM-MSCs), suggesting that MSC populations found across all skeletal tissues share a similar ontogeny [46]. Veritably, a set of minimum criteria have been described for the identification of these cells [47]; although, these criteria still need further refinement as some adult cells have been found to be capable of de-differentiating into stem-like cells in culture, further blurring the line between native MSCs and culture inspired MSC-like cells [48,49]. Regardless, however, both native MSCs and de-differentiated MSC-like cells exhibit demonstrated usefulness for tissue engineering applications as well as stimulating and/or enhancing tissue healing [50,51,52,53]. 

MSCs secrete a large variety of bioactive molecules that facilitate a regenerative environment. This trophic activity deem them immunosuppressive, especially to T-cells, and as a result, allogeneic MSCs can be used for therapeutic purposes [54]. MSCs exhibit significant immunomodulatory capacity and play an important role in maintaining immune homeostasis by interacting with chemokines, cytokines and cell surface molecules [55]. Besides considering which type of MSC is optimal for therapy, another equally important consideration is the microenvironment of the injured tissue in question, which also dictates overall repair success. The local tissue microenvironment can influence how cells integrate into the existing tissue matrix and how they restore the damaged tissue [14]. Tissue structure, organization, extracellular matrix (density and content), cellularity, and vasculature are all determining factors of how quickly and how well it will heal. 

Alongside MSCs, human induced pluripotent stem cells (iPSCs) have also emerged as a potential cell source for skeletal tissue repair. iPSCs are cells that have been dedifferentiated into the state of pluripotency by the induction of select transcription factors including octamer-binding transcription factor-3/4 (OCT3/4), SRY-related high-mobility-group (HMG)-box protein-2 (SOX2), c-MYC, and Kruppel-like factor-4 (KLF4) [56,57]. These cells represent an inexhaustible cell source for tissue repair and regeneration; however, their incredible plasticity leads to concerns about their tendency to differentiate resulting in unexpected and undesirable phenotypic changes, which are now being addressed [58].

## 3. Stimulating Bone Repair

Bone is the most rigid and vital tissue of the skeletal system since it provides our bodies with structure, protects vital organs, and facilitates hematopoiesis occurring in the bone marrow. The structural components that shape the microenvironment of long bones are nanocrystals of hydroxyapatite (referred as mineral phase); collagen type-I (referred as organic phase); cellular components that include osteoclast, osteoblast, and osteocytes; ions (primarily calcium); and lastly, growth factors and cytokines [59]. The most common broken bone injury is the fracture. Compared to the other tissues of the skeletal system, bones are equipped with sophisticated microvasculature regularly supplying necessary components such as nutrients for growth and maintenance of homeostasis. Bone marrow, which is the primary site for hematopoiesis, attracts not only cytokines and growth factors, but it also attracts metastatic cells (in some cancers), hence stability of the bone microenvironment is critical during injury repair and metastatic disease [60].

Although the architecture and microenvironment of bone tissue allows fracture repair to efficiently occur, often healing to pre-injury state, about 10% of fractures will not heal normally [61]. Bone repair after fracture is a complex process involving a series of cellular and molecular events leading to new bone formation through systemic and local factors [59]. In general, fracture healing mainly involves five steps: hematoma formation, inflammation, angiogenesis, fibrocartilaginous callus formation to bony callus formation, and bone remodeling—with stem/progenitor cells contributing to each stage of healing [62,63]. Following bone fracture, the bone marrow is exposed and results in the rapid formation of a hematoma due to damaged blood vessel. This involves an inflammatory response where specific cytokines like interleukin-1 (IL-1), IL-6 and tumor necrosis factor-α (TNF-α) [64], neutrophils, macrophages, and other inflammatory cells initiate the bone healing mechanism, towards endochondral bone formation and remodeling [65]. Growth and differentiation factors like TGF-β superfamily including bone morphogenetic protein (BMPs), as well as insulin-like growth factors (IGFs), fibroblast growth factors (FGFs), and platelet-derived growth factors (PDGF), orchestrate MSC cell proliferation and differentiation (i.e., chondrogenesis, osteogenesis) [66]. Lastly, during revascularization of the injury site (angiogenesis), BMPs stimulate the expression of vascular endothelial growth factor (VEGF) by osteoblasts [67]. Due to these orchestrated events, bones have a high capacity for healing but repairing comminuted fractures that result in bone loss remains a major challenge [68]. Bone is the most commonly transplanted tissue, leading to 1.5 million annual grafts in the United States [69,70]. However, due to hurdles such as the limited availability of donor tissue for grafts [71], donor site morbidity [72], possibility of allograft rejection [73], and ethical dilemmas concerning putting potential donors at risk make this approach challenging [74,75]. Hence, alternative approaches to heal fractured or damaged bone tissue become necessary.

Previous studies have documented the use of cells and other biologics in the treatment of injured bone. Induction of osteogenesis by co-culturing C3H10T1⁄2 mesenchymal stem cells with chondrocytes stimulated well-known bone formation markers including osteopontin, osterix, and osteocalcin, to enhance the bone healing [76,77,78,79]. Other treatment strategies such as osteoanabolic and antiresorptive treatment strategies (including Wnt/ß-catenin signal activation to promote cell proliferation, differentiation, and microcirculation) have also been utilized [80]. Additionally, parathyroid hormone treatment and bisphosphonate injections have had promising results, but they have several limitations since long-term ablation of bone resorption interferes with the ongoing renewal of the bone matrix and affects skeletal integrity, ultimately affecting the fracture healing process [81,82,83,84,85]. These studies collectively suggest that current approaches still need refinement. 

Hematopoietic stem cells (HSCs) have been widely used as a potential candidate for the bone injury healing. Kumar et al. demonstrated using a rat model that the treatment of IGF1 in combination with AMD3100 elevates the growth of fractured bone that is primarily regulated by cell signaling pathways, Akt and Erk [86]. Further, using a clonal cell transplantation model, Malhotra et al. demonstrated that HSCs migrate and give rise to osteocytes and chondrocytes aiding the healing process of fractured bones [87]. Recently, Chan et al. identified human skeletal stem cells (hSSCs) that undergo local expansion upon injury to the bone. Interestingly, upon comparative analysis, the study also identified evolutionary differences between mouse and human skeletogenesis [57]. Furthermore, the periosteum is considered to be the primary source of mesenchymal progenitor cells, giving rise to the fracture callus [88], but there is evidence that BM-MSCs and muscle progenitor cells also contribute to the bone repair process [63,89,90]. 

Exogenous injection/transplantation of MSCs seems to be the most common and most effective avenue of cell-motivated bone fracture repair that has been reported to date. In late 1960, Friedenstein et al. [91,92] first demonstrated the osteogenic potential of BM-MSCs. The authors filled a diffusion chamber with bone marrow and bone marrow fragments, which was placed in filtered culture containers, showing that the ectopic transplantation of bone marrow cells result in osteogenesis [91,92]. These findings were then further validated in the 1980s when it was documented that the injection of bone marrow aspirants directly on to the sites of bone fractures successfully promoted healing [93]. In the past two decades, several preclinical studies documenting the positive role of bone marrow aspirant injections on the healing of fractured bones have been conducted using rodent models [86,94,95]. Clinical studies have also been conducted using bone marrow aspirant to stimulate healing of fractured bones. Independent case studies conducted on 20 patients by Garg et al. and Sim et al. in 1993 showed 90% and 85% radiographic healing of the fractured bones, respectively [96,97]. Kim and colleagues conducted an open randomized clinical trial consisting of 64 patients with a long bone fracture, that were treated with a local injection of 3.0 × 10^7^ osteogenically differentiated autologous BM-MSCs mixed with fibrin [98]. After 2 months, injected patients showed no complications and exhibited significant fracture healing. Overall, these studies demonstrate the efficacy of using BM-MSCs in a clinical setting for bone repair. However, due to the limitations of BM-MSC abundance (especially in older patients [99]) and their reduced regenerative capacity with continuous cell expansion [100], there is a need for a detailed clinical trial considering factors such as age and gender for their therapeutic applications. 

Several groups have shown the potential of exogenous stem cells such as circulating skeletal progenitors as potential therapeutic candidates for the healing of fractured bones. Almost two decades ago, Kuznestove et al. isolated fibroblast-like skeletal progenitors from rodents and humans and demonstrated the significant osteogenic potential of these cells upon transplantation [101]. However, these circulating skeletal progenitors were shown to be rare in humans [102]. Other studies in nude mice have demonstrated that administering human adipose stem/progenitors (ASCs) directly on to sites of calvarial bone injuries induces the expression of osteoblast markers (including Alpl, Col1a1, Runx2) and stimulates bone formation [103,104]. Interestingly, the exogenous cells persisted only for 14–15 days and were eventually replaced by the host’s own cells. 

Progenitor-like cells isolated from dental pulp, referred to as dental pulp stem cells (DPSCs), were also investigated for bone healing due to their potential for differentiating down the osteoblast lineage (in vitro) and their ability to stimulate bone formation (in vivo) [105,106,107]. In 2000, Gronthos et al., first isolated DPSCs from adult human dental pulp, and determined their in vitro and in vivo characteristics [108]. Recently, Fuji et al. demonstrated the osteogenic differentiation of DPSCs in vitro and bone regeneration in vivo in mouse calvaria defects using a derivative of helioxanthin, which is known to induce osteogenic differentiation of pre-osteoblasts and MSCs [108]. Lee et al. compared the osteogenic and bone regeneration potential of DPSCs and BM-MSCs in vitro and in vivo in a rabbit model [109]. Although, DPSCs have been used pre-clinically for repairing various tissues including cardiovascular tissue, cornea, and muscular, further clinical studies are needed to evaluate their therapeutic potential [110,111,112].

Many preclinical studies demonstrate the positive effects of a variety of exogenous and endogenous stem/progenitor cells on bone fracture repair (Table 1). On the other hand, only two ongoing US clinical trials testing the efficacy of human MSCs on bone fracture repair and osteochondral defect repair is being carried out at the time of this review (Table 2) suggesting that there is a fairly large gap that separates bench and bedside. This does not necessarily imply that pre-clinical cell-based strategies for bone repair translate poorly, but rather it is more likely that such strategies are difficult to translate into clinical approaches that comply with established regulatory standards set by the Center for Biologics Evaluation and Research (CBER) branch of the Food and Drug Administration (FDA), which take into careful consideration factors such as cell source, purity/homogeneity, and culturing conditions. Furthermore, this field would benefit greatly from an influx of clinical studies that thoroughly evaluate different factors such as donor age, sex, and the number of cells to be injected as this would improve our understanding of therapeutic applications of exogenous stem/progenitor cells for bone healing.

## 4. Stimulating Tendon and Ligament Repair

Tendon and ligament are the dense connective tissue that connects bone to muscle and bone to bone, respectively. Both are highly prone to injury and can be difficult to repair due to their structural organization, hypo-cellularity and hypo-vascularity [138,139,140,141]. Tendon tissue primarily consists of collagens I, II, III, V, XI, XII, XIV, elastin, and glycosaminoglycans (GAGs) [142,143,144,145]. Type I collagen is a most robust component of the tendon extracellular matrix (ECM) network [146]. Tendon cells, or tenocytes, are fibroblast-like in appearance and they synthesize ECM components including collagen fibers, elastin, proteoglycans [142,147]. The proportion of tendon/ligament ECM constituents can vary among individuals, mainly due to differences in the mechanical loading environment; however, they tend to have relatively consistent physiology and structure [148,149]. 

The healing response to tendon/ligament injury can be divided into three overlapping stages—(1) inflammation, (2) proliferation, and (3) remodeling [150]. These stages are ushered by specific cytokines and cellular processes. In the inflammatory phase, a blood clot is formed after tendon injury, which is followed by activation of chemoattractant inflammatory cells like neutrophils, monocytes, and lymphocytes [151]. The clot primarily serves as a scaffold to harbor the cells and releases important growth factors like TGF-β, IGF-1, PDGF, and VEGF causing inflammation [152]. The second phase, proliferative phase begins roughly after 2 days following injury. This phase is directed by macrophages and tenocytes of the endotenon and epitenon region of the tendon. Macrophages release growth factors to direct cell recruitment and tenocytes help in synthesis of a matrix, primarily consisting of type III collagen [153,154,155]. The proliferative phase is identified by increased cellularity, synthesis of ECM, and the deposition of scar tissue by fibroblasts [156]. After about 1–2 months following injury, the remodeling phase begins with reorganization of tenocytes and collagen fibers, aligning in the direction of stress [156] with a decrease in type III collagen and GAG content [157] and increased synthesis of type I collagen [158]. This process continues for months after injury; however, the newly formed tissue gradually changes to scar-like tendon tissue. The repaired tissue also lacks biomechanical, biochemical, and ultrastructural properties of native uninjured tendon tissue [159,160].

This complex microenvironment of tendon and ligament tissues makes the healing process slow [161,162]. In the US alone, tendon and ligament injuries account for almost half of the 32 million musculoskeletal injuries incurred each year [163]. These rates are rising due to the increasing aged population and also due to increased participation in sports activities [156]. Rotator cuff tears increase with age, from 9.7% in patients 20 years and younger to 62% in patients that are 80 years and older [164], which represents a significant burden [165]. The main challenge in tendon healing is the failure to functionally attach tendon to bone. The attachment, called enthesis, consists of the transitional gradient of tendon, fibrocartilage, calcified fibrocartilage and bone; and it mainly allows dissipation of stress between these tissues of different properties [166,167]. Although the enthesis can be reattached surgically, the gradient is disrupted and replaced with scar tissue exhibiting impaired mechanical properties [160,168]. As with tendon, anterior cruciate ligament (ACL) surgical repair results in failure due to the lack of blood clot formation, intra-articular hypo-vascularity, loss of intrinsic cell migration, and poor healing capacity of ACL [169,170]. There is even a high risk of failure when the ACL is repaired surgically by suturing in adolescent patients [171]. Severe tendon/ligament injuries that result in lost or unsalvageable tissue require tissue autografts, which often lead to donor site morbidity [172,173]. Ultimately, current surgical paradigms fail to restore the functional, biochemical, and structural properties of the native tissue [156]. 

Tenocytes and ligamentocytes (ligament cells) express Scleraxis (Scx)—a transcription factor that regulates the expression of the glycoprotein Tenomodulin, which is a specific marker of these mature cells [174,175,176]. Like other connective tissues, tendon also contains a unique population of heterogeneous tissue-specific MSCs. They are capable of differentiating into tenocytes, and true to the nature of all MSCs, they can differentiate along the chondrogenic, osteogenic, and adipogenic lineages upon in vitro induction [177]. Further, these cells are capable of generating tendon, cartilage, and tendon-bone junction-like tissues in rats [178], rabbits [117], and equine animal models [179]. Over the years, regenerative approaches for treating tendon and ligament injuries have been explored. These include: (i) cell based therapies [180,181,182] (ii) gene therapy [24,183] (iii) orthobiologics/platelet rich plasma injections (PRP) [119,120,184], and recently, (iv) cell free strategies that implement sustained released growth factors to recruit endogenous stem cell that promote healing [121]. Biochemical factors such as cytokines (IL-6, IL-10, IL-1β,TNF-α) [185,186,187] and growth factors (transforming growth factor (TGF)-β, basic fibroblast growth factor (bFGF), IGF-1, PDGF) play a crucial role in maintaining tissue homeostasis [152,188]. Many endogenous and exogenous cell-based therapies have been used to repair tendons and ligaments in pre-clinical models (Table 1).

It has been reported that tendon derived stem/progenitor cells (TDSCs) exhibited a higher regenerative potential towards ruptured Achilles tendon, compared to BM-MSCs at 4 weeks in a rat model [122]. However, due to the limited availability of these cells, they must be culture expanded. Unfortunately, in vitro expansion has been reported to cause the cells to lose their phenotypic markers [189] and studies have demonstrated that the number of TDSCs are greatly reduced with aging, showing diminished proliferative capacity [190,191]. With this in mind, other cell sources are being investigated for therapeutic use.

Bone marrow is a widely explored alternative cell source for tendon and ligament tissue engineering [123,124,125,192]. Gulotta et al. explored the use of BM-MSCs for treating unilateral detachment of the supraspinatus tendon in the rats [193]. Although there were no significant differences found between the treated and untreated groups, they showed that cells were present and metabolically active at the repair site following treatment. The same group found that cell-based strategies alone may not be sufficient to improve the structure, composition, and strength of the healing tendon tissue [193]. Two years later, they showed that administering BM-MSCs transduced with adenoviral-mediated *Scleraxis* improved rotator cuff repair in a rat model [194]. There seemed to be no difference in the histological appearance between the Scx and MSC group, but the Scx transduced cell treated group had more fibrocartilage, higher load-to-failure and stress-to-failure ratio at 4 weeks, compared to BM-MSC group alone. Genetic modifications of administered BM-MSCs and ACL fibroblast with bone morphogenetic factor (BMP)-12 and BMP-13 has also been demonstrated to induce ligamentogenic differentiation, in vitro [195]. 

ASCs are also interesting for tissue engineering due to their accessibility and great abundance when extracted from human subcutaneous adipose tissue [196,197]. Park et al. demonstrated that rat ASCs, when treated with growth differentiation factor-5 (GDF-5), exhibited enhanced ECM production and tendonogenic differentiation of cells, in vitro [198]. Multiple studies have reported the in vivo efficacy of using ASCs to stimulate tendon healing and improve biomechanical properties and normal collagen fiber organization, compared to the control groups [126,199,200]. Recently, Kokubu et al. demonstrated that ASCs improved tendon healing by stimulating collagen fiber reformation and preventing ectopic ossification of tendons in mice, compared to the control group at 2 and 4 weeks after injury [201]. This study suggested that ASCs can modulate inflammation and induce neovascularization at the site of injury. Tracking of transplanted ASCs revealed that they were present at 2 and 7 days post transplantation, but no longer present by 3 weeks, post transplantation [201].

When used in combination with fibrin sealant or hydrogels, ASC treatment has been reported improved tendon healing with increased expression of Col1, Scx, and Tenomodulin in the damaged tendon tissue. In vivo survival of ASCs injected with scaffolds (fibrin sealant or hydrogel) into the defect region was confirmed at day 14 [127] and day 31, post injury [202]. While these studies effectively demonstrate that there are several different approaches for utilizing exogenous cell treatments (with/without bioactive scaffolds or growth factors) to stimulate tendon and ligament repair [119,120,127,183,184,202], further comprehensive studies are needed to assess the safety and efficacy of these approaches to take the research from bench-to-bedside.

Endogenous cell-based approaches for tendon regeneration have also emerged as a promising strategy consisting of applying different growth factors and biomaterials to effectively recruit native stem/progenitor cell population. Solaiman et al. demonstrated a tissue engineering integrated approach, utilizing both in vitro and in vivo models, by a precise spatiotemporal growth factor delivery system embedded in 3D-printed flexible scaffolds, which resulted in regeneration of the tendon-to-bone interface by recruitment of endogenous stem/progenitor cells [121]. Immunofluorescence analysis 7 days post-implantation showed the infiltration of CD146^+^ cells into the growth factor embedded scaffold, suggesting an endogenous cell source does exist for tendon-to-bone healing in a rat model [121]. The regenerative potential of tissue-resident stem/progenitor cells (CD146^+^) in a rat model was evaluated by Lee et al., [128] These cells, after enriching with connective tissue growth factor (CTGF), were mixed with fibrin gel and delivered at the injury site. By the end of week 1 and 2, CTGF led to a dense, aligned collagen fibers, compared to the group without CTGF delivery. By post-operative week 4, CTGF generated tendon exhibited dense collagen structure compared to the scar-like tissue in the fibrin-alone group [128]. Furthermore, expression of different growth factors are evaluated in early phases of tendon healing [203,204]. 

Several ongoing clinical studies are trying to better understand the implications of treating injuries with MSCs alone or in combination with bioactive scaffolds to repair tendon and ligament (Table 2). Hernigou et al. showed 10-year follow up results, after injecting MSCs to have enhanced rate of healing and reduced number of re-tears over time [205]. Kim et al. revealed that an injection of ASCs in combination with fibrin sealant, significantly improved re-tear rates of rotator cuff injuries [206]. Ligament injuries like ACL tears have also shown promising results in small number of patients using autologous injection of bone marrow nucleated cells [207]. Murray et al. developed a bridge enhanced ACL repair (BEAR) which combines suture repair with an extra cellular matrix scaffold to bridge the gap between the ligament ends [27] and presented the first-in-human study to show no graft or repair failures following a two year follow up [28].

## 5. Stimulating Articular Cartilage Repair

Articular cartilage is a resilient yet flexible tissue which is a vital component of the skeletal system. The extracellular matrix (ECM) of articular cartilage is mainly composed of water, proteoglycans, and collagen fibers. The chondrocyte is the primary type of cell found in cartilage, which almost completely lacks blood vasculatures and neural architecture [208]. This means that the sole mode of nutrient supplements to the chondrocytes is though adjacent tissues and fluids such as subchondral bone and synovial fluid, respectively [209,210]. In comparison to other connective tissues, articular cartilage has the lowest turnover of ECM and it has a very limited capacity for healing after injury [211]. Chondrocytes reside in small compartmented cavities called lacunae that are surrounded by large areas of dense cartilage ECM making it difficult for these cells to move freely or migrate in response to biological cues of damaged tissue. Moreover, the lack of blood vasculature prevents the infiltration of articular cartilage by circulating immune cells [212]. Multiple approaches have been documented to help restore cartilage without surgical proceedings, but the fact remains that there are virtually no clinical treatment options in existence that completely restore damaged articular cartilage to its native state [213,214,215,216,217]. 

Given its poor capacity for healing, knee injury resulting in cartilage damage is a great clinical challenge [218,219,220,221,222]. Widely practiced clinical procedures for attempting cartilage restoration include endogenous cell-based methods like bone marrow stimulation by creating microfractures [223,224], and exogenous transplantation of tissue and cells such as the use of osteochondral grafts [225,226], and autologous chondrocyte implantation (ACI) [227,228,229,230,231,232]. ACI was first described by Brittberg and colleagues in 1994 where patients’ own cartilage tissues were harvested in one surgery to extract the chondrocytes, followed by cell culture expansion and injection into the defect site during a second surgery [231]. This treatment, along with the matrix induced autologous chondrocyte implantation (MACI) method [233], has shown positive clinical results for larger cartilage defects [234,235]. However, each method has its own limitations that have been previously discussed to great lengths [236,237,238,239,240].

With regards to novel methodologies that are still in development for cartilage repair, pre-clinical strategies fall into one (or both) of two avenues: (1) utilizing biomaterials that can replace/fill lost cartilage and restore some loadbearing capacity to defect region; (2) utilizing/stimulating cells to assist with rebuilding lost cartilage ECM [241]. Cellular approaches involve BM-MSCs and ASCs along with the induction of chondrogenesis. Chondrogenesis is thought to be more functional in a 3D culture system as chondrocytes tend to lose their original characteristics, dedifferentiate in the monolayer and acquire a fibroblastic morphology [242,243]. Studies have demonstrated the chondrogenic differentiation of BM-MSCs and ASCs in monolayer culture as well as in 3D aggregate cultures [40,45,244,245,246]. The preclinical use of stem/progenitor cells for cartilage repair has been documented in different animal models (Table 1). Ko et al. investigated the chondrogenic and hypertrophic potential of human iPSCs compared to BM-MSCs and also demonstrated the healing of damaged cartilage in rats by grafting iPSCs [130]. Additionally, Xing et al. demonstrated that a single injection of human umbilical cord derived MSCs into sprague dawley rat knee joint, 4 weeks post-surgery, resulted in a significant retardation of OA progression compared to controls [131]. Other studies have also reported that stem cells induce cartilage repair in rodents [132,133], porcine [133] as well as the equine models [134]. Although cell/tissue transplantation-based therapeutic strategies seem promising, there are drawbacks such as donor-site morbidity, the possibility of graft rejection and rapid cell ‘wash-out’ from synovial fluid [247]. Due to these limitations, scientists are actively considering the guided delivery of the biologics such as proteins, nucleic acid, and growth factors to redirect the native/endogenous cells to the site of cartilage tissue injury [248,249,250]. These strategies are intended to motivate cartilage stem/progenitor cells (CPCs) and mature chondrocytes to help accelerate healing [251]. 

For large cartilage defects, growth factors are used in combination with biomaterials/scaffolds that have the dual role of providing the necessary structural support while also facilitating the recruitment of endogenous cells over time [135]. Scaffold can also be laced with other bioactive molecules, like chemokines, to promote cartilage repair. Chen et al. demonstrated a silk fibroin-porous gelatin scaffold (capable of sustained release) loaded with growth factors stromal-derived factor-1α (SDF-1α) and TGF-β1 can promote in vitro MSC homing, migration and chondrogenesis and cartilage regeneration in vivo [136]. Other bioactive molecules like IGF-1 and FGF-1 encapsulated in bio-scaffolds, were reported to accelerate the repair process ex vivo and in vivo [137,252,253,254,255].

Current clinically practiced methods to reduce pain, increase joint lubrication, and subside inflammation include: intra-articular injections of platelet rich plasma (PRP), hyaluronic acid, and corticosteroids. Administering specific growth factors have also showed promise for stimulating healing [248,249]. An early phase clinical trial [26] seem to suggest that intra-articular injections of ASCs induce a degree of cartilage regeneration, as they can differentiate along the chondrogenic lineage [256,257,258]. However, ASCs maintain their chondrogenesis potential up to 15–16 passages with diminishing efficiency to differentiate into chondrocytes, comparatively less than that of BM-MSCs [246,259]. A clinical trial comparing the efficacy of using microfracture vs. application of ASCs on a collagen scaffold (NCT02090140) for articular cartilage defect repair is currently ongoing. Simultaneously, randomized phase III clinical trials are being conducted to evaluate which source of stem cells are superior (NCT03818737). In addition, ongoing is a trial comparing the outcomes in patients with osteoarthritis (OA) injected with fragmented adipose tissue (NCT03467919) in comparison to the current standard treatment of treating with corticosteroid to alleviate pain. Refer to Table 2 for more details on ongoing clinical trials.

Although studies to date suggest that the stem/progenitor cells are capable of cartilage repair, the combination approach of using cells with different growth factors and bio-scaffolds may be beneficial for future pre-clinical studies seeking to improve tissue repair efficacy. While facilitating the migration of endogenous cells to areas of injury is clinically appealing from a regulatory standpoint, aging ensures that these cells have dwindling capacity for self-renewal and healing. Unfortunately, elderly individuals make up the largest population suffering from cartilage degeneration. Taking the road less traveled by genetically modifying these cells, in order to enhance their regenerative potential, may change the therapeutic paradigm for cartilage repair.

## 6. Conclusions

Skeletal tissues like bone, cartilage, tendon, and ligament are difficult to repair completely upon injury due to their complex microenvironment. Surgical approaches to repair these damaged tissues are widely used but due to their limitations, cell-based approaches have emerged, and they are being tested in both pre-clinical models and in current ongoing clinical trials. The approach of stimulating recruitment of endogenous cells to the injury site is preferable and advantageous in order to avoid donor site morbidity and graft/cell rejection that is associated with use of exogenous cell-based approaches detailed here. Unfortunately, in the aging population, the existing native stem/progenitors may have diminished capacity for proliferation and repair, hence the use of exogenous cells may be preferable. An intriguing solution that should be more deeply explored in the future is to combine these approaches—by administering both exogenous cells and growth factors that will also help recruit native cells to aid in repair. 

## Figures and Tables

**Table 1:** Pre-clinical studies of endogenous and exogenous use of stem/progenitor cells in skeletal tissue.

Tissue	Approach	Animal Model	Injury Model	Experimental Treatment	Outcomes/Results	References
Bone	Endogenous	Mice	Long Segmental Defect	Growth factor + AMD3100 treatment	2 weeks: IGF1 showed increased proliferation and migration of isolated MSC as well as augmented bone growth.	Kumar et al., [86] 2012
Bone	Exogenous	Nude mice	Calvarial defect (4 mm)	Undifferentiated Human ASCs + PLGA scaffold + (rh) BMP2	8 weeks: Gross analysis, microCT, and histological examination showed complete healing and trabecular bone formation in the hASCs treated group compared to the scaffold only group and empty defects.	Levi et al., [103] 2010
Bone	Exogenous	Rats	Femoral fracture	BMSCs + skin fibroblasts	5 weeks: Callus size and mechanical properties were significantly higher in the MSC injected group compared to fibroblast and the PBS control. Quantitative analysis showed GFP-positive MSCs were present in callus in MSC group at 5 weeks after fracture.	Huang et al., [113] 2015
Bone	Exogenous	Mice	Femur fracture	Mouse BMSCs/RFP	42 days: BMSCs injected on day 7 post-fracture accelerated fracture healing with improved callus and bone quality.	Wang et al., [114] 2018
Bone	Exogenous	Rat	Bone nonunion	Primary MSCs sheet + SDF1 injection	4 and 8 weeks: At 4 weeks, new formed bone tissue united the distal and proximal sites in the MSC sheet/SDF group compared to 5 other groups. At 8 weeks, the MSC sheet/SDF group showed complete bridging of the fracture site, forming hard bony union.	Chen et al., [115] 2016
Bone	Exogenous + Endogenous	Mice	Osteogenesis impairment	Peptidomimetic ligand (LLP2A) + Alendronate (LLP2A-Ale) injection	3 and 12 weeks: At 3 weeks, the hMSC cells injected intravenously in the xenotransplantation model were observed at the bone surface in the LLP2A-Ale group. At 12 weeks, the LLP2A-Ale group could augment bone formation in mice.	Guan et al., [116] 2012
Tendon	Exogenous	Rats	Multi-differentiation potential	TSCs + Matrigel (gel-cells)	8 weeks: Transplantation of TSCs subcutaneously resulted in the formation of tendon, cartilage and bone-like tissues.	Zhang et al., [117] 2010
Tendon	Exogenous	Rat	Achilles Tendon	MSCs cultured in hypoxic and normoxic condition	2 and 4 weeks: Tendon rupture site and biomechanical properties were superior in hypoxic MSC group compared to the normoxic and control.	Huang et al., [118] 2013
Ligament	Endogenous; Growth factors	Canine	ACL defect	Collagen-Platelet Rich Plasma (PRP) Scaffold	3 and 6 weeks: The percent filling defect was significantly higher in the treated group at both 3 and 6 weeks compared to the untreated defects. Mechanically, the ACL treated group had 40% increase in strength at 6 weeks, compared to untreated defects.	Murray et al., [119] 2006
Ligament	Endogenous; Growth factors	Porcine	ACL defect	Suture + Collagen-Platelet Rich Plasma (PRP) hydrogel	4 weeks: At 4 weeks, the collagen-PRP hydrogel group stimulated healing and improved biomechanical properties after suture repair, compared to suture repair alone. However, both groups remained significantly inferior to the intact ligament group.	Murray et al., [120] 2007
Tendon	Endogenous; Growth factors	Rat	Rotator Cuff	3D printed scaffold + Growth factors (CTGF, CTGF + TGF-b + BMP2)	1 and 4 weeks GF embedded (+GF) scaffolds promoted recruitment of endogenous tendon progenitor cells and healed tendon-to-bone via formation of cartilaginous interface compared to –GF scaffold.	Solaiman et al., [121] 2019
Tendon	Exogenous	Rat	Achilles Tendon	TDSCs and BMMSCs	1, 2 and 4 weeks: TDSCs showed higher regenerative potential with high mechanical strength, better appearance density and well-organized longitudinal fibrous structure and BMSCs also showed positive results.	Al-Ani MK et al., [122] 2015
Tendon	Exogenous	Rabbit	Patellar Tendon defect	BMSCs + Type I bovine collagen gel	4 weeks: Mechanically and histologically, the MSC + gel group showed significantly greater material and structural properties compared to the collagen gel alone control. However, treatment group improvements were not impressive compared to the normal healthy tendon.	Awad et al., [123] 1999
Ligament	Exogenous	Rabbit	ACL Reconstruction	BMSCs + Silk scaffold	8, 16 and 24 weeks: The MSC/Scaffold group showed abundant ligament ECM (Col I was more prominent compared to Col III and Tenascin-C), compared to the scaffold alone control. The tensile strength was comparable to the mechanical properties of daily activities.	Fan et al., [124] 2008
Tendon	Exogenous	Rat	Rotator Cuff injury	BMSCs + PRP	4 and 8 weeks: Gene and protein detection at 4 weeks, showed that combined therapy enhanced the expression of growth factors and genes related to tendon repair (Col I, Tenomodulin, Scx). At 8 weeks, mechanical testing demonstrated that combined therapy was most efficient to promote tissue regeneration, compared to single therapy control (PRP alone and MSC alone).	Han et al., [125] 2019
Tendon	Exogenous	Rat	Tendon injury	hASC + fibrin glue	4 weeks: Treatment group of hASCs demonstrated enhanced tendon healing biomechanically, compared to the fibrin alone and sham group. Cells were showed to survive for 4 weeks, in vivo and secreted human-specific Col I and Tenascin-C.	Lee et al., [126] 2017
Tendon	Exogenous	Rat	Partial Transection of Achilles Tendon	ASCs + Fibrin Sealant (FS) from serpent venom	21 days: In vivo analysis at day 14 revealed higher quantification of the transplanted fluorescent ASCs in the tendon treated with ASCs + FS compared to ASC alone. The ASCs group up-regulated Tenomodulin expression compared to normal (without transection), transection alone and the FS group. TIMP-2 and Scx expression compared to N group. FS group demonstrated great organization of collagen fibers followed by ASCs + FS and ASCs alone in comparison to N	Frauz et al., [127] 2019
Tendon	Endogenous; Growth factor	Rat	Patellar Tendon	TSCs (CD146^+^) + Fibrin glue + CTGF	1, 2 and 4 weeks: CTFG treated CD146^+^ cells led to tendon regeneration with dense collagen fibers, compared to the untreated CD146^+^. By week 4, the CTGF group generated tendon with dense collagen fibers compared to the fibrin alone group and tensile property on the level of native tendon compared to CD146^-^ and untreated CD146^+^.	Lee et al., [128] 2015
Tendon	Endogenous; Growth factor	Sheep	Rotator Cuff injury	rhPDGF-BB coated sutures	6 weeks: rhPDGF-BB coated sutures enhanced histologic scores of sheep rotator injury and enhanced tendon healing. However, load to failure was equivalent to standard suture repair.	Uggen et al., [129] 2010
Cartilage	Exogenous	Rat	Osteo-chondral Defect	hiPSCs pellet or hiPSCs + alginate hydrogel	12 weeks: Defects treated with chondro-induced hiPSCs implantation had smooth, firm tissue with good restoration of articular surface compared to control or alginate alone. However, histological appearance showed reduced amount of proteoglycan compared to the normal cartilage.	Ko et al., [130] 2014
Cartilage	Exogenous	Rat	Osteoarthritis	Human umbilical MSCs + Hyaluronic acid (HA)	6 and 12 weeks: Macroscopic observation of the femur surface at 6 weeks, showed signs of OA progression with cartilage surface roughness and osteophyte formation compared to preserved cartilage in MSC + HA group; at 12 weeks, joint surface showed OA progression in all 3 groups. Histologically at 6 weeks, the MSC + HA group showed abundant proteoglycan and reduced cartilage loss, whereas at 12 weeks, Saf-O staining was significantly reduced compared to 6 weeks Hence, single injection of hUC-MSCs had temporary effects to decelerate OA progression.	Xing et al., [131] 2020
Cartilage	Exogenous	Rat	Full thickness cartilage defect (2mm)	BMSCs + SUMO1/SUMO2,3/SUMO1,2,3	4 weeks: BMSCs overexpressing SUMO1 differentiated into articular cartilage with hard surface; BMSCs overexpressing SUMO1,2 reduced inflammation and improved damaged cartilage microenvironment; BMSCs overexpressing SUMO1,2,3 showed better survival, less inflammatory response, and improved tissue repair.	Liu et al., [132] 2020
Cartilage	Exogenous	Rabbit and Minipigs	Osteo-chondral defect	ECM group: autologous MSC-derived ECM scaffold; BMS group: Bone marrow stimulation	Rabbits: 6hrs, 3 and 7 days: Macroscopic appearance of exudate healing wounds showed less fibrosis and histology showed evenly distributed chondrocyte in the EMS group compared to the BMS. The CFU-F assay showed increased number of bone MSCs in the ECM group. Minipigs: 6 months: Macroscopic and MRI finding improved in the ECM compared to BMS group. Repaired tissue in ECM had similar histological characteristic to normal hyaline cartilage.	Tang et al., [133] 2019
Cartilage	Exogenous	Rat	Full thickness cartilage defect (2 mm)	Equine BMSCs and Synovial Fluid-Derived MSC (SFMSCs) + agarose gel	1 and 12 weeks: At 1 week, the knee joint showed the presence of MSCs at the injured site.Macroscopic and histological analysis demonstrated better healing of cartilage in MSC treated knees at 12 weeks, compared to the control. SFMSC treated showed significantly higher Col II, suggesting presence of hyaline cartilage at the defect site.	Zayed et al., [134] 2018
Cartilage	Endogenous; Growth factors	Rabbit	Humeral Head incision	TGF-β3 adsorbed or TGF-β3-free + collagen hydrogel	4 months: The TGF-β3 treated group had significantly greater matrix and articular cartilage thickness compared to the TGF-β3-free group, showing that the articular cartilage of the synovial joint was regenerated by homing endogenous cells. The TGF-β3 treated group also had consistent distribution of Col II and Aggrecan.	Lee et al., [135] 2010
Cartilage	Endogenous; Growth factors	Rats	Osteo-chondral Defect (1.6mm)	Silk fibroin scaffold + SDF-1α + TGF-β1	12 weeks: Scaffold treated with + SDF-1α and TGF-β1 (GSTS) had the most significant cartilage regeneration compared to 4 other control groups. The GSTS group also produced more type II collagen compared to other groups, which generated fibrocartilage.	Chen et al., [136] 2019
Cartilage	Endogenous; Growth factors	Rabbit	Osteo-chondral Defect (5 mm)	Hydroxyapatite collagen (Hap/Col) scaffold + FGF-2 with 10 and 100 µg/mL concentration collagen (HAp/Col) scaffold	3,6, 12 and 24 weeks: Abundant bone formation observed in the Hap/Col group compared to the defect group. The FGF10 group demonstrated abundant bone regeneration as well as satisfactory cartilage regeneration with a hyaline-like appearance.	Maehara et al., [137] 2010

**Table 2:** List of few ongoing clinical trials for skeletal tissue.

Condition	NCT Identifier	Title	Status	Intervention
Non Union Fracture	NCT03325504	A Comparative Study of 2 Doses of BM Autologous H-MSC + Biomaterial vs Iliac Crest AutoGraft for Bone Healing in Non-Union	Recruiting	Biological: Cultured Mesenchymal Stem Cells Procedure: Autologous iliac crest graft
Osteochondral Fracture of Talus	NCT03905824	The Effectiveness of Adding Allogenic Stem Cells After Traditional Treatment of Osteochondral Lesions of the Talus	Recruiting	Biological: Allogenic stromal mesenchymal cells derived from the umbilical cord Procedure: Debridement and microfracture
Full Thickness Rotator Cuff Tear	NCT02484950	Mesenchymal Stem Cell Augmentation in Patients Undergoing Arthroscopic Rotator Cuff Repair	Recruiting	Biological: Mesenchymal stem cell augmentation in rotator cuff repair Procedure: Standard arthroscopic rotator cuff repair
Rotator Cuff Tear Rotator Cuff Tendinitis	NCT03752827	Autologous Adult Adipose-Derived Regenerative Cell Injection into Chronic Partial-Thickness Rotator Cuff Tears	Recruiting	Device: Adipose Derived Regenerative Cells Drug: Corticosteroid
Rotator Cuff Tear	NCT03688308	Bone Marrow Derived Stem Cells for the Treatment of Rotator Cuff Tears	Recruiting	Procedure: Arthroscopic rotator cuff repair with bone marrow aspirate concentrate
Rotator Cuff Tear	NCT03551509	LifeNet: Extracellular Matrix Graft in Rotator Cuff Repair	Recruiting	Biological: ArthroFLEX ECM scaffold graft Procedure: Control Biological: Crossover
Rotator Cuff Tears	NCT04325789	Rotator Cuff Healing Using a Nanofiber Scaffold in Patients Greater Than 55 Years	Recruiting	Device: Rotium nanofiber graft
ACL—Anterior Cruciate Ligament Rupture	NCT03294720	BioACL Reconstruction with Amnion Collagen Matrix Wrap and Stem Cells Case Series	Active, not recruiting	Procedure: Bio-ACL Device: amnion wrap and BMAC
ACL—Anterior Cruciate Ligament Rupture	NCT03294759	Bio ACL Reconstruction Amnion Collagen Matrix Wrap and Stem Cells	Active, not recruiting	Other: Bio ACL Other: Control
Anterior Cruciate Ligament Tear	NCT02664545	Bridge-Enhanced ACL Repair vs. ACL Reconstruction	Active, not recruiting	Device: BEAR Scaffold Procedure: Tendon Graft
Defect of Articular Cartilage Cartilage Injury Osteoarthritis, Knee	NCT02696876	Synovium Brushing to Augmented Microfracture for Improved Cartilage Repair	Recruiting	Device: Arthroscopic synovial brushing Procedure: Microfracture
Degenerative Lesion of Articular Cartilage of Knee	NCT02090140	Microfracture Versus Adipose Derived Stem Cells for the Treatment of Articular Cartilage Defects	Recruiting	Procedure: ADSC Application Procedure: Microfracture
Osteoarthritis, Knee	NCT04205656	Prospective Evaluation of PRP and BMC Treatment to Accelerate Healing After ACL Reconstruction	Recruiting	Biological: Leukocyte-Poor Platelet Rich Plasma (LP-PRP) Biological: Bone Marrow Concentrate (BMC) Other: Control group (Placebo)
Osteoarthritis, Knee	NCT02805855	Autologous Culture Expanded Mesenchymal Stromal Cells for Knee Osteoarthritis	Recruiting	Drug: Autologous Adipose-Derived Mesenchymal Stromal Cells
Knee Osteoarthritis	NCT03014401	The Effect of Adipose-Derived Stem Cells for Knee Osteoarthritis	Recruiting	Procedure: Arthroscopic debridement with stem cell transplantation Procedure: Arthroscopic debridement only
Osteoarthritis, Knee Knee Pain	NCT03467919	The Effect of Micro Fragmented Adipose Tissue (MFAT) on Knee Osteoarthritis	Recruiting	Procedure: Micro Fragmented Adipose Tissue Procedure: Corticosteroid injection
Post-Traumatic Osteoarthritis of Knee	NCT04222140	Early Regenerative Intervention for Post-Traumatic Osteoarthritis	Not yet recruiting	Combination Product: ERIPTO Protocol Biological: BMAC Only
Knee Osteoarthritis	NCT04043819	Evaluation of Safety and Exploratory Efficacy of an Autologous Adipose-derived Cell Therapy Product for Treatment of Single Knee Osteoarthritis	Active, not recruiting	Drug: PSC-01
Musculoskeletal Pain Knee Osteoarthritis Cartilage Injury Cartilage Degeneration	NCT03477942	Impact of Mesenchymal Stem Cells in Knee Osteoarthritis	Recruiting	Biological: Autologous Mesenchymal Stem Cells
Articular Cartilage Disorder of Knee Articular Cartilage; Degeneration	NCT03101163	Efficacy and Safety Study of Intra-Articular Injections of Autologous Peripheral Blood Stem Cells Following Subchondral Drilling Surgery for the Treatment of Articular Cartilage Injury in the Knee	Recruiting	Biological: Autologous peripheral blood stem cells and hyaluronic acid Other: Hyaluronic acid
Osteoarthritis, Hip	NCT03608579	Autologous Culture Expanded Adipose Derived MSCs for Treatment of Painful Hip OA	Recruiting	Drug: Autologous Adipose Derived Mesenchymal Stromal Cells
Osteoarthritis	NCT03818737	Multicenter Trial of Stem Cell Therapy for Osteoarthritis (MILES)	Recruiting	Biological: Autologous Bone Marrow Concentrate (BMAC) Biological: Adipose-derived Stromal Vascular Fraction (SVF) Biological: Umbilical Cord Tissue Drug: Depomedrol and Normal saline (Corticosteroid injection)
